# Individual and Environmental Determinants of Provider Continuity Among Urban Older Adults With Heart Failure: A Retrospective Cohort Study

**DOI:** 10.1177/2333721418801027

**Published:** 2018-09-20

**Authors:** Miriam Ryvicker, David Russell

**Affiliations:** 1 Visiting Nurse Service of New York, New York City, USA; 2 Appalachian State University, Boone, NC, USA

**Keywords:** continuity of care, chronic conditions, environmental factors

## Abstract

**Objective:** Continuity in patient–provider relationships is important to providing high-quality care for older adults with chronic conditions. We investigated individual and environmental determinants of provider continuity for office-based physician visits among urban older adults with heart failure. **Method:** We linked Medicare claims with data on neighborhood characteristics for a retrospective cohort of community-dwelling Medicare beneficiaries with heart failure in New York City (*N* = 50,475). **Results:** Mean continuity using the Bice–Boxerman index was 0.33 (*SD* = 0.22) (possible range of 0 [no continuity] to 1 [perfect continuity]). Multivariable regression indicated that provider continuity was higher among older, female, and dually eligible beneficiaries. Those with more chronic conditions had higher continuity, controlling for number of medical specialties seen. Continuity was lower for beneficiaries in neighborhoods with high median income and high primary care density. **Conclusion:** Individual and environmental predictors of provider continuity among urban older adults with heart failure could help to identify those at risk of care fragmentation.

## Introduction

Maintaining a continuous patient–provider relationship is considered a key element in the quality of care for older adults, especially those with chronic conditions. Older adults who maintain a continuous relationship with a single health care provider (i.e., those with provider continuity) experience fewer medication errors and preventable hospitalizations, utilize fewer unnecessary medical procedures, and incur lower health care costs compared with those with low provider continuity (Amjad, Carmichael, Austin, Chang, & Bynum, 2016; Bayliss et al., 2015; Cheng & Chen, 2014; Hussey et al., 2014; Nyweide et al., 2013; Romano, Segal, & Pollack, 2015). Receiving care from the same provider over time facilitates care coordination by improving communication and management of patient problems (Christakis, Wright, Zimmerman, Bassett, & Connell, 2003). Conversely, care fragmentation—where a patient’s care is split across multiple providers in primary and/or specialty care—is a source of inefficiency in the U.S. health care system, leading to higher overall utilization and greater risk of hospitalizations, especially among older adults (Agha, Frandsen, & Rebitzer, 2017).

Establishing provider continuity may be especially important for patients with heart failure, which affects 6.5 million adults in the United States (Benjamin et al., 2018). Individuals with heart failure tend to have frequent hospitalizations, multiple comorbidities, and complex medication regimens that present challenges for effective care management (Foust, Naylor, Bixby, & Ratcliffe, 2012; Jencks, Williams, & Coleman, 2009; Mosterd & Hoes, 2007; Murtaugh et al., 2017; Naylor et al., 2004; O’Connor et al., 2016). Heart failure patients may consult with multiple clinicians, including primary care physicians, hospitalists, and specialists—which together may lead to greater care fragmentation across providers (Wagner, Schaefer, Horner, Cutsogeorge, & Perrault, 2011). Similarly, older adults with multiple chronic conditions face an increased risk of disability and death compared with those with fewer conditions and may be more likely to be evaluated by numerous providers (Salive, 2013; Tinetti, Fried, & Boyd, 2012).

Lapses in provider continuity have been established as a risk factor for poor health care outcomes among older adults with chronic conditions (Amjad et al., 2016; Cheng & Chen, 2014; Nyweide et al., 2013). However, few studies have examined the factors that influence provider continuity. Identifying individual and environmental determinants of continuity could help to identify patients at heightened risk for fragmented care and its associated adverse outcomes. This study aims to address this gap by examining associations of individual and contextual factors with provider continuity among older Medicare beneficiaries with heart failure living in New York City (NYC). We expand upon prior research on the health effects of neighborhood environmental factors such as socioeconomic status, the built environment, and the health care environment.

Although previous studies have examined the influence of these contextual factors on health behavior and service utilization, their effects on provider continuity are understudied. A large body of research has examined the effects on health behavior of the local built environment, including measures of “walkability” such as the presence of sidewalks, access to public transportation, the mix of residential and commercial land use, and traffic safety (Lovasi, Hutson, Guerra, & Neckerman, 2009; Rundle et al., 2007). Among older adults, greater walkability has been linked to greater physical activity (Berke, Koepsell, Moudon, Hoskins, & Larson, 2007), walking for errands (King, 2008), greater lower-extremity strength (Michael, Gold, Perrin, & Hillier, 2011), and lower blood pressure (Li, Harmer, Cardinal, & Vongjaturapat, 2009). Moreover, attributes of the health care environment, such as the supply of health care, influence health care accessibility and utilization (The Center for Evaluative Clinical Sciences, 1996; Continelli, McGinnis, & Holmes, 2010). In addition, environmental characteristics including poor transportation infrastructure, inadequate medical services, and remoteness to treatment centers may present barriers to health care access, including the ability to see the same provider over time (Mechanic & Tanner, 2007; Prentice, 2006; Russell, Oberlink, Shah, Evans, & Bassuk, 2018; Stahler et al., 2007; Stahler, Mennis, Cotlar, & Baron, 2009). This study examines the effects of environmental factors on provider continuity using a uniquely configured dataset comprised of Medicare claims linked with geographic data sources for an urban population of older adults with heart failure.

## Method

### Study Design

This retrospective cohort study used data on a sample of community-dwelling Medicare fee-for-service beneficiaries in NYC aged 65 and older. Beneficiary data for the years 2008 through 2010 were acquired in 2012 through a Data Use Agreement from the Centers for Medicare and Medicaid Services (CMS) as part of a larger study investigating the influence of neighborhood environmental factors in health care access and outcomes among chronically ill older adults. Beneficiary addresses were linked to geographic data on neighborhood socioeconomic composition, public transit access, and primary care supply.

### Individual-Level Data and Measures

Beneficiary data included Medicare enrollment information, demographics, and claims for services provided under Medicare fee-for-service during 2008-2010. As a proxy for low income, a binary indicator for dual eligibility for Medicare and Medicaid was defined as whether the beneficiary was Medicaid-eligible for at least 1 month during 2008. Binary variables for selected chronic conditions, including depression and Alzheimer’s disease/related dementias, were defined using CMS’s Chronic Condition Warehouse (CCW) indicators (Chronic Condition Data Warehouse, 2015). A count variable was created from the CCW to represent an individual’s total number of chronic conditions. We derived a count variable for noninstitutionally based “evaluation and management” (E&M) visits under Medicare Part B from 2008 through 2010 (CMS, 2016). E&M codes are used for visits that offer routine screening and management of chronic conditions, occurring in outpatient “offices” (e.g., private physician offices, hospital outpatient departments, or clinics). We also calculated the total number of individual providers seen for E&M visits, as well as a count of the unique specialty types of providers seen for E&M.

Provider continuity was measured with the Bice–Boxerman index (Amjad et al., 2016; Bice & Boxerman, 1977; Romano et al., 2015). This index reflects the relative share of a beneficiary’s E&M visits that were conducted by distinct physicians during the study period; scores range from 0 (each visit was conducted by a different physician) to 1 (all visits were conducted by the same physician). The calculation of continuity scores was limited to beneficiaries with four or more E&M visits to ensure that the scores were valid and meaningful.

### Geographic Data and Measures

Geographic variables were derived from publicly available data sources, using the most recent available data prior to the time frame of the Medicare data. U.S. Census (2000) data (U.S. Census Bureau, 2002) were used at the census tract level to measure neighborhood median income and access to public transportation (based on the proportion of respondents who used public transit to get to work). We used a measure of primary care supply from the Primary Care Service Area (PCSA) Project (2007) of the Dartmouth Institute. PCSAs represent geographic approximations of markets for primary care services (Goodman et al., 2003). We assigned beneficiaries to a PCSA based on the zip code of residence (which is nested within a PCSA), totaling 52 PCSAs in our dataset. We used Dartmouth’s age- and sex-adjusted measure of the number of primary care providers (PCPs) per 100,000 residents at the PCSA level. For modeling purposes, we divided the aforementioned geographic measures into quartiles. In addition to these measures, we derived a binary indicator of the availability of a Federally Qualified Health Center (FQHC) at the zip code level using publicly available data (Health Resources and Services Administration, 2010).

### Analytic Sample

The sample drew from the full universe of Medicare beneficiaries who were aged 65 and older as of January 1, 2008, and lived in NYC’s five boroughs. We selected individuals for the current analysis if they (a) were community-dwelling, defined as having no days in a skilled nursing facility or other nonhospital inpatient facility during the 3-year study period (2008-2010); (b) had no months of Medicare HMO coverage during the study period, because managed care claims are not available in the data; (c) had a heart failure diagnosis according to the CCW indicator; and (d) had an address that successfully matched to the census tract. The match rate for addresses was 96%; there were no notable biases in geographic distribution by borough comparing those that did and did not match. The resulting matched sample included 50,475 individuals dispersed throughout 2,103 census tracts across NYC’s five boroughs.

### Statistical Analysis

Descriptive statistics were used to examine individual characteristics and service use for the overall sample and across groups of beneficiaries with low, medium, and high provider continuity. These comparison groups were based on tertiles within the continuity distribution (Amjad et al., 2016). We used multilevel (mixed effects) regressions to examine individual and environmental factors associated with provider continuity (the dependent variable), specifying “crossed” random effects because an individual is assigned to different types of geographic units that are not nested. Construction of variables and descriptive analysis were performed in SAS Version 9.3 (SAS Institute, 2011). Geocoding was performed using the Geosupport Desktop Edition™ software Version 11.4 (NYC Department of City Planning, 2012). Multilevel regression models were run in R Version 3.1.2 using the “lme4” package (Bates, Maechler, Bolker, & Walker, 2015). All study procedures were approved by the Institutional Review Board of the Visiting Nurse Service of New York.

## Results

### Individual Characteristics, Neighborhood Environments, and Service Use

The characteristics of Medicare beneficiaries and their neighboring environments are shown in Table 1. The average beneficiary age was 78 years (*SD* = 7.2), with the greatest proportion between ages 75 and 84 (45%). The majority of beneficiaries were female (61%) and non-Hispanic White (68%). A large share of beneficiaries included in the study were dually-eligible (47%). Study beneficiaries had an average of 7 chronic conditions (*SD* = 2.3). The most prevalent comorbidities included hypertension (85%), diabetes (54%), rheumatoid arthritis or osteoarthritis (51%), chronic kidney disease (20%), and chronic obstructive pulmonary disease (16%).

**Table 1.** Characteristics of NYC Medicare Beneficiaries with Heart Failure by Provider Continuity Score (*N* = 50,475).

	Continuity Index Score^a^			
	Overall (*N* = 50,475)	Low (*n* = 16,823)	Medium (*n* = 16,831)	High (*n* = 16,821)
Age, mean (*SD*)	78.4 (7.2)	77.4 (6.7)	78.5 (7.1)	79.4 (7.6)
Age category, %				
65-74	33.4	38.0	33.0	29.1
75-84	45.3	46.2	45.4	44.2
85+	21.4	15.8	21.6	26.7
Female, %	60.5	57.7	59.0	64.9
Race, %				
White	67.6	74.9	69.2	58.7
Black	12.2	8.0	11.9	16.6
Hispanic	11.8	10.1	11.1	14.3
Asian/Pacific Islander	4.9	3.3	4.3	7.0
Other/Unknown	3.5	3.7	3.5	3.4
Dual eligible, %	47.2	50.0	44.7	46.7
Number of chronic conditions, mean (*SD*)	7.0 (2.3)	7.6 (2.3)	7.0 (2.2)	6.3 (2.2)
Chronic condition, %				
Diabetes	54.3	56.1	55.1	51.8
Hypertension	85.0	86.6	85.0	83.5
Chronic kidney disease	20.4	23.2	20.9	17.0
COPD	15.9	18.4	16.0	13.2
Cancer	10.9	13.8	11.4	7.4
Alzheimer’s disease or related dementia	15.7	16.2	14.5	16.3
Depression	13.3	18.7	12.2	9.1
Rheumatoid arthritis or osteoarthritis	50.9	62.7	50.4	39.6
Environmental characteristics, mean (*SD*)				
Median income (in thousands of dollars)	53.1 (33.6)	55.3 (37.1)	54.0 (33.5)	49.9 (29.7)
Proportion using public transit	0.51 (0.15)	0.50 (0.15)	0.50 (0.15)	0.51 (0.15)
Primary care physician density at PCSA level	61.5 (18.6)	64.0 (19.3)	61.3 (18.4)	59.1 (17.8)
Proportion of zip codes with FQHC available	0.44 (0.50)	0.43 (0.49)	0.44 (0.50)	0.47 (0.50)
Health service use in 2008-2010				
No. E&M visits, mean (*SD*)	51.8 (38.5)	68.8 (44.4)	50.7 (33.2)	36.1 (29.0)
No. E&M visits, median	42	59	43	28
No. physicians seen for E&M visits, mean (*SD*)	9.4 (6.8)	15.0 (7.5)	8.5 (4.0)	4.5 (2.8)
No. specialties seen for E&M visits, mean (*SD*)	6.5 (3.6)	9.4 (3.4)	6.4 (2.5)	3.7 (2.1)
Provider continuity index, mean (*SD*)	0.33 (0.22)	0.14 (0.04)	0.26 (0.05)	0.58 (0.19)

*Note.* COPD = chronic obstructive pulmonary disease; PCSA = primary care service area; FQHC = Federally Qualified Health Center; E&M = evaluation and management.

a Continuity index scores were ordered and grouped into tertiles.

The average median income of census tracts where beneficiaries lived was US$53,102, and about half (51%) of neighborhood residents used public transit to get to work. An average of 62 PCPs (*SD* = 18.6) served each of the PCSAs where study beneficiaries lived. FQHCs were available in 44% of beneficiaries’ zip codes.

Beneficiaries received an average of 52 E&M visits (*SD* = 38.5) during the 3-year period by nine unique physicians (*SD* = 6.8) across seven specialties (*SD* = 3.6). The average provider continuity score was 0.33 (*SD* = 0.22).

### Bivariate Relationships Between Individual and Neighborhood Characteristics and Provider Continuity

Also shown in Table 1, we examined bivariate (unadjusted) relationships between level of provider continuity (low, medium, and high tertiles) and characteristics of the individual and their environment. Low continuity included values of 0 through 0.19; medium included 0.20 through 0.35; and high included values above 0.35 through 1. Compared with those with low and medium continuity, the high continuity group was older (mean age 79 compared with 77 in the low group), had a lower proportion of dually eligible individuals (47% vs. 50% in the low group), and had a greater proportion of female beneficiaries (65% vs. 58% in the low group). On average, beneficiaries with high continuity had fewer chronic conditions (mean of 6.3 compared with 7.6 in the low group) and saw fewer types of specialists (mean of 3.7 vs. 9.4 in the low group). Individuals with high continuity lived in areas with lower median income (roughly US$50,000 compared with US$55,000 for the low group) and lower PCP density (mean of 59.1 PCPs compared with 64.0 for the low group).

### Regression Examining Predictors of Provider Continuity

The multilevel mixed-effects regression examining individual and environmental predictors of provider continuity is shown in Table 2. The model indicates that, controlling for other factors, older age (β = .0218; *p* < .0020; age 85+ vs. age 65-74) and being female (β = .0147; *p* < .0001) was associated with higher continuity. Compared with Whites, continuity scores were lower among Hispanics (β = −. 0092; *p* < .0001) and higher for Asians (β = .0353; *p* < .0001). Controlling for the number of specialties seen and other covariates, beneficiaries with more chronic conditions had higher continuity (6-8 conditions [β = .0063; *p* < .0001]; 9+ conditions [β = .0071; *p* < .0001]). However, those with a diagnosis of depression (β = −.0096; *p* < .0001) had lower continuity compared with beneficiaries without depression. No relationship was observed with Alzheimer’s disease/related dementias ( β = −.0013; *p* = .5304). A key driver of continuity was the number of specialty types seen; the top three quartiles for number of specialty types showed significantly lower provider continuity compared with the lowest quartile (*p* < .0001).

**Table 2.** Multilevel Regression Examining Predictors of Provider Continuity (*N* = 50,475).

	Estimate	*SE*	*p* value
Individual-level measures			
Female	0.0147	0.0015	<.0001
Age category (ref = 65-74)			
Age 75-84	0.0108	0.0016	<.0001
Age 85+	0.0218	0.0020	<.0001
Dual eligible	0.0000	0.0018	.9839
Race (ref = White)			
Black	0.0007	0.0027	.7818
Hispanic	−0.0092	0.0027	<.0001
Asian/Pacific Islander	0.0353	0.0036	<.0001
Other/Unknown	0.0043	0.0039	<.0001
Number of chronic conditions (ref = 1-5)			
6-8 chronic conditions	0.0063	0.0018	<.0001
9+ chronic conditions	0.0071	0.0023	<.0001
Depression	−0.0096	0.0022	<.0001
Alzheimer’s/related dementia	−0.0013	0.0021	.5304
Number of specialty types seen (ref = Quartile 1)			
Number of specialties: Quartile 2	−0.2397	0.0021	<.0001
Number of specialties: Quartile 3	−0.3377	0.0021	<.0001
Number of specialties: Quartile 4	−0.4255	0.0023	<.0001
Environment-level measures			
Median income (ref = Quartile 1)			
Median income: Quartile 2	0.0027	0.0027	.3229
Median income: Quartile 3	0.0017	0.0030	.5749
Median income: Quartile 4	−0.0092	0.0033	.0053
Proportion using public transit (ref = Quartile 1)			
Use of public transit: Quartile 2	0.0024	0.0028	.3951
Use of public transit: Quartile 3	0.0014	0.0030	.6533
Use of public transit: Quartile 4	−0.0010	0.0032	.7532
Primary care density at PCSA level (ref = Quartile 1)			
Primary care density: Quartile 2	−0.0082	0.0064	.2072
Primary care density: Quartile 3	−0.0101	0.0065	.1280
Primary care density: Quartile 4	−0.0176	0.0067	.0109
FQHC available at zip code level	−0.0027	0.0021	.1914

*Note.* Ref = reference group; PCSA = primary care service area; FQHC = Federally Qualified Health Center.

Relationships between environmental characteristics and provider continuity were mixed. Beneficiaries residing in neighborhoods in the top income quartile had lower provider continuity (β = −.0092; *p* = .0053) compared with those residing in the lowest income quartile. Neighborhood public transit use and proximity to an FQHC were both unassociated with provider continuity. Beneficiaries who lived in areas with the highest PCP density had lower continuity scores ( β = −. 0176; *p* = .0109) compared with those living in areas with the lowest PCP density.

### Sensitivity and Stratified Analyses

In addition to the results shown, we conducted sensitivity analyses removing individuals who were in the top 5% of the distribution of total E&M visits. The results did not change substantially after removing these outliers. We also ran stratified models according to the number of chronic conditions, to examine whether predictors of continuity varied for individuals with different levels of multimorbidity. The major findings were similar across the stratified models.

## Discussion

This study examined individual and environmental determinants of provider continuity in a community-dwelling sample of older adults with heart failure in NYC. Overall, the distribution of continuity scores in our sample was consistent with findings reported in prior studies of continuity using the Bice–Boxerman index in the Medicare population (Amjad et al., 2016; Romano et al., 2015). Key drivers of continuity included the interplay of multimorbidity and the number of specialty types seen by the individual. In bivariate analyses, having more chronic conditions was associated with lower continuity. However, this relationship changed when controlling for the number of specialties seen. In multivariable regression, individuals with more chronic conditions had higher continuity, while seeing more specialties was associated with lower continuity. This suggests that the bivariate relationship between the number of chronic conditions and continuity was driven partly by the fact that those with more comorbidities tend to see more specialists. This is consistent with prior research on multimorbidity as a driver of service utilization (Schiltz et al., 2017; Whitson et al., 2016).

Beneficiaries with depression tended to have lower continuity compared with those without depression. This finding is consistent with recent research documenting that adults with psychiatric conditions are more likely to report frequently changing their usual place of care (Weissman et al., 2017; Weissman, Russell, Beasley, Jay, & Malaspina, 2016). Promoting better integration of mental health treatment and primary care may help to improve continuity for this population (Weissman et al., 2017).

Age was also a driver of continuity. That increasing age was associated with greater continuity may be interpreted alongside psychological theories of social behavior, which suggest that older adults are more likely than younger adults to favor long-term relationships within limited social networks over newer and more diverse social connections (Carstensen, Isaacowitz, & Charles, 1999; Löckenhoff & Carstensen, 2004). Prior research suggests that older adults tend to have long-standing relationships with physicians, with a third of their relationships spanning 10 years or more (Weiss & Blustein, 1996).

Among the environmental factors considered, we found that individuals living in higher-income neighborhoods and in areas with greater physician density had lower continuity. This finding is consistent with some previous studies, while running counter to others. For instance, one study found that higher-income urology patients were more likely to change physicians (DuGoff, Bekelman, Stuart, Armstrong, & Pollack, 2014), while a study of urban children found that those living in low-income neighborhoods had lower ambulatory care continuity (Mustard, Mayer, Black, & Postl, 1996).

It is possible that neighborhood income operated as a proxy for unmeasured characteristics related to individual service use behavior. For example, perhaps individuals living in higher income and/or more physician-dense areas have greater health literacy and skills in navigating the health care system, which may lead to more “shopping around.” Although doctor shopping has been studied in relation to specific factors such as drug-seeking behavior (McDonald & Carlson, 2013), mental illness (Norton et al., 2011), and obesity (Gudzune, Bennett, Cooper, Clark, & Bleich, 2014), the full range of reasons underlying doctor shopping remains unknown (Sansone & Sansone, 2012). It is possible that doctor shopping may be influenced by contextual factors in high-supply geographic locations. In addition, given that social networks have been shown to influence service utilization (Czapka & Sagbakken, 2016; Goldman & Cornwell, 2015; Pullen, Perry, & Maupome, 2018), the social networks of individuals living in areas with greater financial and health care resources could be more conducive to seeking care from multiple providers. Whether these fragmented patterns are detrimental for individual outcomes in the context of more affluent, resource-dense areas is a question for further investigation.

Some study limitations should be noted. First, the claims data lack information on potential factors in health care utilization such as social support, function, cognition, education, and psychosocial measures. Future work could examine these factors using data sources that link claims with survey data, such as the National Health and Aging Trends Study (NHATS; Johns Hopkins School of Public Health & Westat, 2015). However, using national survey data would limit the more granular geographic linkages that are possible when examining a large sample within a selected city.

Second, the findings may have been affected by sampling bias. Our prior research among chronically ill Medicare beneficiaries suggested that Blacks, Hispanics, and dually eligible beneficiaries were more likely to have lapses in E&M visits (Ryvicker & Sridharan, 2018). Thus, the required minimum of four E&M visits during the 3-year study period introduced selection bias to the analytic sample, which we confirmed in exploratory analysis. The bias remained when relaxing the criteria to a 2-visit minimum. Although we used a well-established continuity measure (Amjad et al., 2016; Bice & Boxerman, 1977; Romano et al., 2015), future research should explore alternative measurement approaches.

Third, our NYC sample contains an unusual degree of heterogeneity at both the individual and environmental levels; generalizability of study findings to other geographic areas may be limited. Future research could examine determinants of provider continuity in a national sample (Johns Hopkins School of Public Health & Westat, 2015) or in other specific geographies, including urban, rural, and suburban areas. Fourth, potential selection bias into neighborhoods is a common concern in research on neighborhood effects (Diez Roux, 2004); future analyses could address this with an instrumental variable approach (Fish, Ettner, Ang, & Brown, 2010).

Finally, there have been significant developments in health care policy since the study time frame. While our data offer baseline findings on determinants of continuity among urban, chronically ill Medicare beneficiaries, future research should examine data since enactment of the Affordable Care Act, especially given that this population tends to have high service use and expenditures (Hayes et al., 2016; Hussey et al., 2014).

## Conclusion

This study offers insight into the individual and environmental factors associated with variations in provider continuity among urban older adults with heart failure. Heart failure is associated with frequent hospitalizations and transitions within and across provider settings (Foust et al., 2012; Naylor et al., 2004; O’Connor et al., 2016). Frequent care transitions pose risks for heart failure patients—in the form of critical communication lapses, medication errors, polypharmacy and other problems (Foust et al., 2012)—that could potentially be mitigated by continuous oversight and coordination by a designated provider. Future research could examine the correlates of provider continuity in populations with other complex chronic conditions, especially those with a heightened risk for hospitalizations, such as diabetes, chronic obstructive pulmonary disease, and dementia. Moreover, it would be fruitful to expand analysis to other geographic areas and examine trends in provider continuity over time. This work could help to raise awareness among clinicians who may be positioned to improve care coordination for patients at risk of care fragmentation, especially older adults with multiple chronic conditions.

Prior research has examined the causes and consequences of care fragmentation and has proposed potential systemic and policy solutions to promote greater continuity and care coordination (Agha et al., 2017; Mate & Compton-Phillips, 2014; Stange, 2009). These solutions include but are not limited to a shift toward payment structures that incentivize the integration of care across settings, implementation of cross-setting electronic health records, and decreasing reliance on specialty care (Mate & Compton-Phillips, 2014; Stange, 2009). While the causes of and potential solutions for care fragmentation appear to be situated within the health care system, our findings suggest that external patient-level and environmental factors may influence a person’s unique susceptibility to care fragmentation. As policy makers and providers continue to work toward systemic solutions, tailored care coordination interventions may also be needed for older adults with multimorbidity and other vulnerable populations.
